# Transient cavity dilatation during supine exercise bicycle stress testing: mechanistic insights

**DOI:** 10.1186/s44156-026-00105-7

**Published:** 2026-03-02

**Authors:** B. L. Elliott, J. C. Flynn, A. Macnab, M. Stout, K. Pearce, L. E. Dobson

**Affiliations:** 1 Department of Medicine, University of Manchester, Manchester, UK; 2 Department of Cardiology, Manchester Foundation NHS Trust, Manchester, UK

**Keywords:** Exercise stress echocardiography, Left ventricular cavity dilatation, Supine bicycle, Myocardial ischaemia, Hypertensive response to exercise, Non-obstructive coronary artery disease, Diastolic dysfunction, Chest pain

## Abstract

**Background:**

Cavity dilatation is occasionally observed during supine bicycle exercise stress echocardiography (SBSE). The underlying mechanisms are poorly understood.

**Aims:**

This study aimed to characterise patients with left ventricle (LV) cavity dilatation and a decrease in the left ventricular ejection fraction (LVEF) during SBSE.

**Methods:**

A total of 653 patients who underwent SBSE were evaluated. Those with exercise-induced cavity dilatation (defined as increased cavity size and a decrease in LVEF) were evaluated (*n* = 29). A control group (*n* = 37) of patients with a hypertensive response and a normal ESE was also evaluated.

**Results:**

A total of 33/653 (5.1%) patients had an abnormal LV cavity response to exercise, with 15/29 (51.7%) having significant underlying coronary artery disease (CAD). Comparisons were made between patients with CAD (*n* = 15) and those with nonobstructive coronary arteries (NCAs, *n* = 14). NCA patients had significantly higher peak diastolic blood pressure (DBP) (NCA-CD 109 ± 17 mmHg vs. CAD-CD 96 ± 16 mmHg, *p* = 0.044) and rate-pressure product (RPP) (NCA-CD 28,623 ± 4474 vs. CAD-CD 23,649 ± 4763, *p* = 0.007). There was a trend toward increased dyspnoea at peak exercise in NCA (NCA-CD 35.7% vs. CAD-CD 6.7%, *p* = 0.080), and CAD patients showed a higher observed frequency of severe chest pain (CAD 53.3% vs. NCA 14.3%, *p* = 0.050). When patients with NCA and cavity dilatation were compared with a control group of patients with a hypertensive response to exercise but no cavity dilatation, no significant differences were observed.

**Conclusion:**

Cavity dilatation is observed in 5% patients undergoing SBSE and is attributable to significant CAD in approximately half of patients. NCA cavity dilatation is associated with increased RPP and DBP at peak exercise, with a trend toward increased dyspnoea. Severe chest pain at peak exercise was observed more frequently in CAD patients.

## Introduction

Exercise stress echocardiography is a well-validated test for the investigation of suspected coronary artery disease (CAD) [1, 2]. Exercise stress echocardiography can be performed using a supine bicycle or treadmill. Supine bicycle exercise stress echocardiography (SBSE) is often preferred to treadmill exercise echocardiography (TME), as images can be taken during exercise [3].

Research suggests that SBSE improves the visualisation of regional wall motion abnormalities (RWMAs), thus increasing the sensitivity of detecting CAD [3, 4]. There are, however, differences in the cardiovascular demands of the two techniques, with SBSE leading to an attenuated heart rate response but a more marked increase in blood pressure than treadmill exercise does [4]. Patients on SBSE seem to achieve a longer duration of exercise but lower metabolic equivalents (METs) and peak rate-pressure products (RPPs) than those on TME [4].

**Fig. 1:**
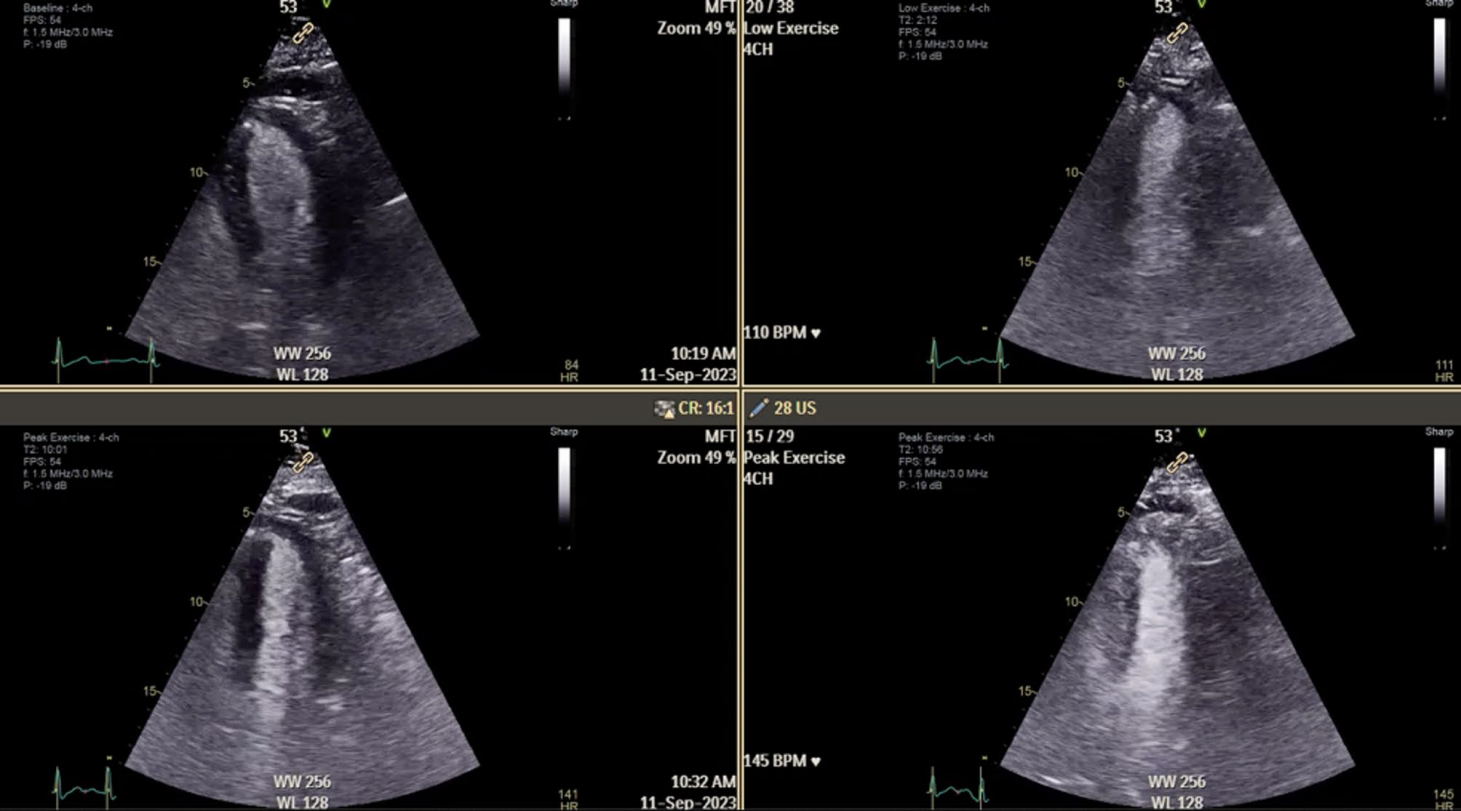
A normal left ventricular response to exercise; a reduction in the LV systolic cavity size and an increase in the LV ejection fraction with increasing workload. The top left image shows the LV at rest, the top right image shows the LV at low workload, the bottom left image is at the intermediate stage, and the bottom right image was taken during peak exercise. LV: left ventricle

A normal and healthy response to exercise is a reduction in the left ventricle (LV) cavity size, accompanied by a corresponding increase in the left ventricular ejection fraction (LVEF) of 10% to meet increased oxygen demand [1, 5] (Fig. 1). This phenomenon is often referred to as the LV contractile reserve; a healthy myocardium should exhibit increased contractile force in response to stress [6].

If cavity dilatation and/or a decrease in LVEF is observed during exercise, this is considered an abnormal response. The underlying mechanisms of this response are multiple and poorly understood. Multivessel CAD can be causative in this setting due to widespread ischaemia of multiple territories and is an important differential [1, 7–9].

**Fig. 2:**
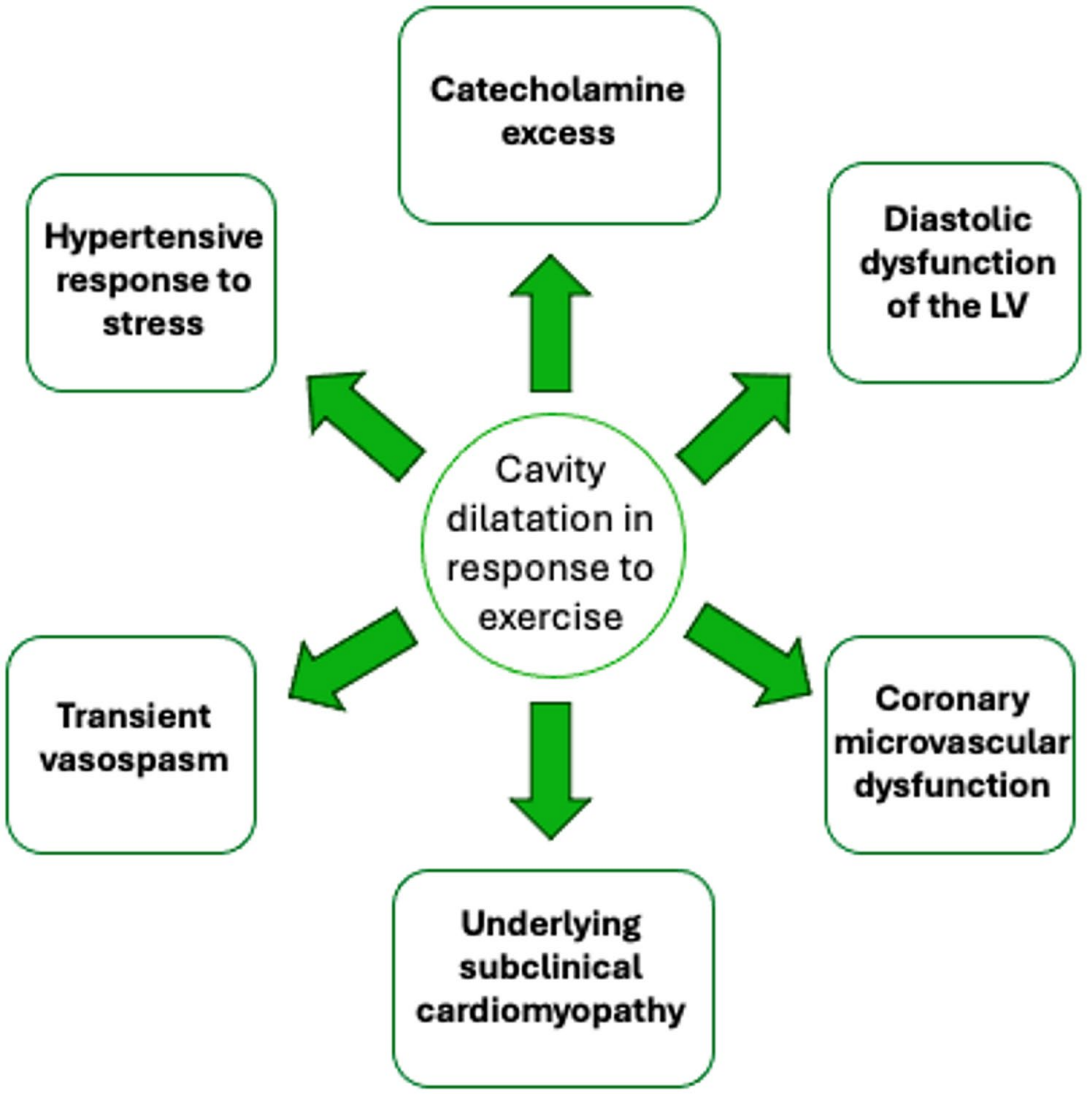
Proposed mechanisms behind an abnormal LV response (cavity dilatation and/or a reduction in LVEF) during exercise. Figure adapted from references [2, 9, 12–14]. LV= Left ventricle, LVEF= left ventricular ejection fraction

Notably, some patients respond to stress with widespread hypokinesis, LV cavity dilatation and a decrease in LVEF, despite investigations revealing unobstructed coronary arteries [10, 11]. Several mechanisms have been postulated in the literature including a hypertensive stress response, catecholamine excess, underlying subclinical cardiomyopathy/diastolic dysfunction and coronary microvascular dysfunction [2, 12–14]. The proposed mechanisms are illustrated in Fig. 2.

The aim of this study was to characterise patients with cavity dilatation in response to exercise, aiming to determine whether any specific characteristics are associated with this abnormal response.

## Methods

### Patient selection

A total of 653 consecutive patients from March 2022 to April 2024 who underwent physiological SBSE performed at a single tertiary centre (Wythenshawe Hospital, Manchester, UK) were retrospectively analysed. Patient indications for SBSE were in line with national guidelines. Patients were identified via electronic patient records (EPIC systems, Verona, Wisconsin, USA) and Intellispace echo archiving software (Philips, Best, Netherlands).

**Fig. 3:**
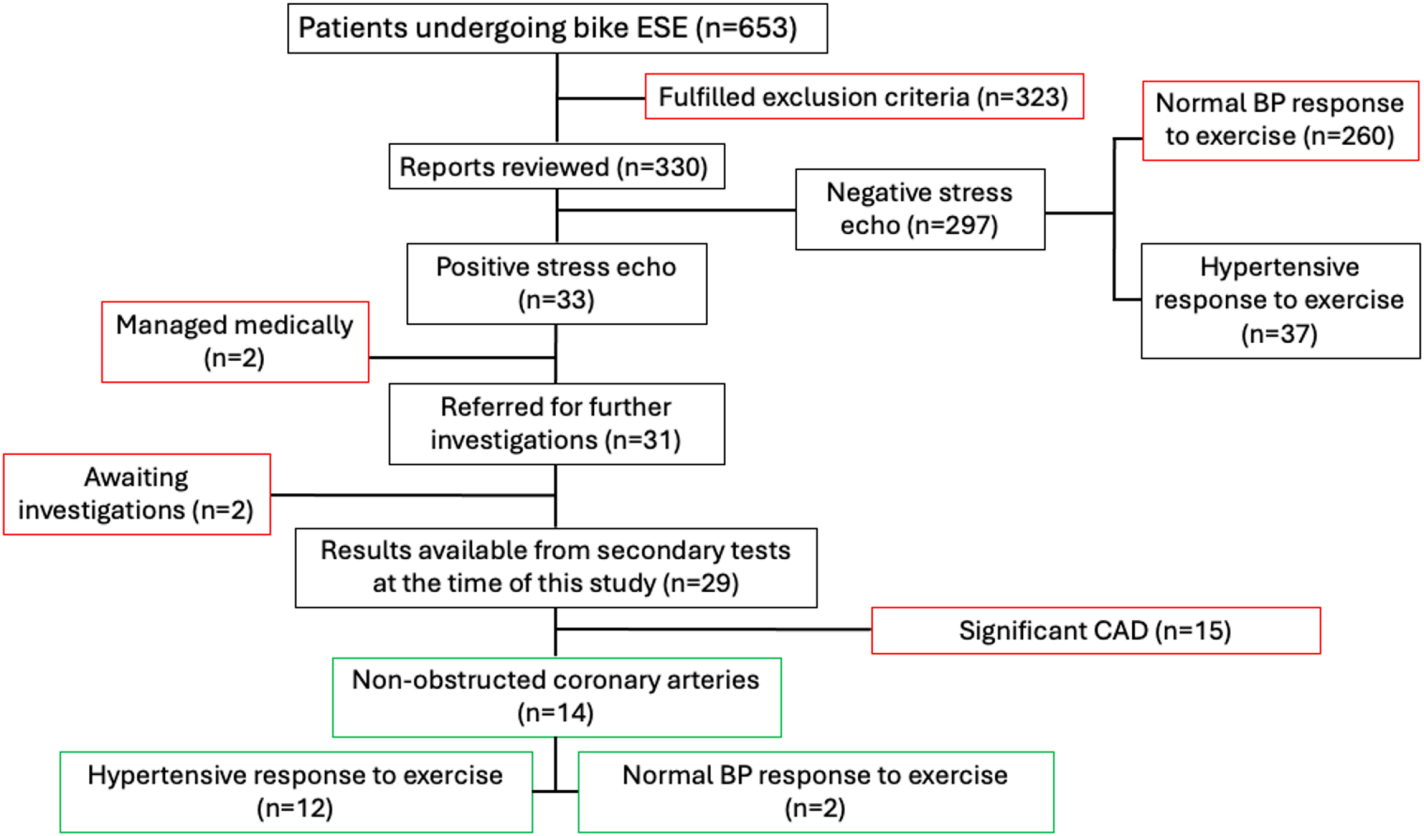
A flow diagram presenting the screening process. The text boxes outlined in red represent patients excluded, and the text boxes in green represent the main study population with cavity dilatation. ESE = exercise stress echocardiography, CAD= coronary artery disease, BP= blood pressure

Patients were excluded if they met the following criteria: (i) $\:\ge\:$ moderate valvular disease, (ii) previous percutaneous coronary intervention (PCI), myocardial infarction (MI), previous coronary artery bypass graft (CABG), (iii) history of CAD, (iv) history of arrhythmias, (v) premature cessation of testing, or vii) history of heart failure. The SBSE reports of 330 patients were analysed (Fig. 3). A positive SBSE for cavity dilatation was defined as a decrease in LVEF and an increase in end-systolic cavity size on visual assessment by a board-certified cardiologist with experience in stress echocardiography. A control group of patients with a hypertensive response to stress but normal left ventricular response to exercise were also evaluated.

Patient characteristics were recorded including age, sex, body mass index (BMI), smoking status, family history (FH) of CAD, hypertension (HTN), dyslipidaemia, type 2 diabetes mellitus (T2DM) and a past medical history (PMH) of anxiety or depression. Non-flow-limiting CAD was defined as coronary stenosis of $\:\le\:$50% or normal epicardial coronary arteries following either computed tomography coronary angiography (CTCA) or invasive coronary angiography. RPP was calculated by multiplying heart rate (HR) by the systolic blood pressure (SBP) for both patients at rest and at peak stress. RPP was used to estimate myocardial oxygen consumption, and thus indirectly measure cardiac workload [15, 16].

### Bike stress echocardiography protocol

SBSE was performed using a supine bicycle and Vivid E Series Cardiovascular Ultrasound system (GE healthcare, Illinois, Chicago). Patients were asked to stop beta-blockers or non-dihydropyridine beta-blockers for 48 h prior to testing. A WHO bike ergometer protocol was used [12] (Table 1) with a constant cadence (between 50 and 60 rpm). The criteria for test termination included the development of severe symptoms (dyspnoea, intolerable leg fatigue or severe chest pain), significant wall motion abnormalities, significant arrythmias or a severe hypertensive response defined as an SBP > 220 mmHg and diastolic blood pressure (DBP) > 120 mmHg. In the absence of any criteria for test termination, patients were exercised until exhaustion.

**Table 1:** The standard supine bicycle exercise protocol was as follows [12]

Time (minutes)	0	2	4	6	8	10	12	14	16
Standard (Watts)	25	50	75	100	125	150	175	190	190

Blood pressure and HR were measured at baseline, during the initial 25 W workload, at peak exercise, and during recovery. Three-channel cardiac rhythm monitoring was performed continuously via the echocardiogram machine. Prior to each test, comprehensive baseline images obtained via conventional two-dimensional echocardiography were acquired and evaluated. This included measurements of LV and right ventricle (RV) size, LV wall motion abnormalities, LV and RV function, aortic root size and aortic valve opening. Assessment of the need for contrast agent was considered if ≥ 2 LV segments could not be visualised. The echocardiographic images obtained at each stage of exercise (baseline, initial 25 W workload, peak exercise, and recovery) included parasternal long-axis, parasternal short-axis (mid-ventricular), and apical 4-, 3- and 2-chamber views. Visual assessment of LV cavity size and global systolic response at peak exercise was performed in accordance with established stress echocardiography practice [17]. All echocardiograms were analysed by a senior echocardiographer who had experience of interpreting over 2,000 stress echocardiograms.

### Statistical analyses

Statistical analyses were performed via Excel software (Microsoft, Redmond, WA, USA). Continuous variables are presented as mean ± standard deviation and were compared between groups using Welch’s t-test, which does not assume equal variances and is recommended for small to moderate sample sizes. Fisher’s Exact Test was used for categorical variables due to small sample sizes. For all the data, statistical significance was defined as *p* < 0.05.

## Results

In total, 33/653 (5.1%) patients who underwent SBSE were identified with exercise-induced cavity dilatation. Among these 33 patients, 4 patients had missing data and were unable to be further evaluated. Twenty-nine patients with cavity dilatation were studied. The mean age of the 29 patients evaluated was 61.4 ± 8.3 years, and 48.3% of these patients were female. The average BMI was 27.8 ± 4.9 m^2^ and 58.6% of patients were on hypertensive therapy. The overall patient characteristics of this group are shown in Table 2.

**Table 2:** Patient characteristics of all study participants with cavity dilatation (*n* = 29)

Patient characteristics	Mean ± SD
Age (years)	61.4 ± 8.3
BMI (kg/m^2^)	27.8 ± 4.9
	**% (n)**
Number of females	48.3 (14)
Number on antihypertensive medication	58.6 (17)
Type 2 diabetes	20.7 (6)
Dyslipidaemia	48.3 (14)
Smoker or ex-smoker	62.1 (18)
Family history of CAD	62.1 (18)
PMH of anxiety or depression	37.9 (11)

### Follow-up evaluation

51.7% (15/29) of cavity dilatation patients were subsequently found to have obstructive CAD (CAD-CD), defined as $\:\ge\:50\%$ coronary artery stenosis in a major vessel. 60% (9/15) of the CAD-CD patients were referred for coronary revascularisation, either a CABG or PCI.

12/14 (85.7%) of patients with non-obstructed coronary arteries and cavity dilatation (NCA-CD) exhibited a hypertensive BP response to exercise.

### Subgroup analysis

#### CAD-CD vs. NCA-CD

Table 3 presents the clinical characteristics and stress echocardiography findings for patients with cavity dilatation during SBSE, comparing CAD-CD (*n* = 15) to patients with NCA-CD (*n* = 14).

**Table 3:** Patient characteristics and bike stress echocardiography parameters in patients with cavity dilatation, comparing patients with underlying coronary artery disease (CAD-CD) to those with nonobstructed coronary arteries (NCA-CD)

Variable	CAD-CD (*n* = 15)	NCA-CD (*n* = 14)	*p* value
**Patient characteristics**			
Age (years)	63 ± 8.2	59.5 ± 7.8	*p* = 0.249
Females, % (n)	33.3 (5)	64.3 (9)	*p* = 0.143
BMI, kg/m^2^	28.5 ± 5.1	27.2 ± 4.7	*p* = 0.481
Antihypertensive medication, % (n)	60 (9)	57.2 (8)	*p* = 1.000
Type 2 diabetes, % (n)	26.7 (4)	14.3 (2)	*p* = 0.651
Dyslipidaemia, % (n)	60 (9)	35.7 (5)	*p* = 0.272
Smoker or ex-smoker, % (n)	60 (9)	64.3 (9)	*p* = 1.000
FH of CAD, % (n)	60 (9)	64.3 (9)	*p* = 1.000
PMH of anxiety or depression, % (n)	33.3 (5)	42.9 (6)	*p* = 0.710
**Echocardiography parameters**			
Resting LV systolic dysfunction, % (n)	20 (3)	14.3 (2)	*p* = 0.684
Resting HR	83 ± 20	77 ± 13	*p* = 0.345
Peak HR	128 ± 16	138 ± 14	*p* = 0.084
Change in HR	44 ± 61	61 ± 14	*p* = 0.310
Resting SBP	161 ± 27	149 ± 31	*p* = 0.278
Peak SBP	186 ± 34	208 ± 27	*p* = 0.064
Change in SBP	26 ± 32	59 ± 33	*p* = 0.011*
Resting DBP	87 ± 11	78 ± 14	*p* = 0.067
Peak DBP	96 ± 16	109 ± 17	*p* = 0.044*
Change in DBP	9 ± 13	31 ± 20	*p* = 0.001*
Resting RPP	13,086 ± 2431	11,479 ± 2925	*p* = 0.121
Peak RPP	23,649 ± 4763	28,623 ± 4474	*p* = 0.007*
Exercise time (mins: secs)	06:43 ± 03:12	07:48 ± 02:59	*p* = 0.354
**Symptoms during test**			
Presence of any symptoms, % (n)	66.7 (10)	64.3 (9)	*p* = 0.893
Chest pain, % (n)	60 (9)	28.6 (4)	*p* = 0.089
Dyspnoea, % (n)	6.7 (1)	35.7 (5)	*p* = 0.080
Severe chest pain, % (n)	53.3 (8)	14.3 (2)	*p* = 0.050

Data are expressed as the mean ± standard deviation or as a proportion of the population (%), with n = the number of patients. * indicates statistically significant results (*p* < 0.05)

There was no significant difference in patient characteristics or CAD risk factors between these groups. Females accounted for the majority of the population with NCA-CD at 64.3%. The average age for patients with CAD-CD was 63 ± 8.2 years, whereas it was 59.5 ± 7.8 years for patients with NCA-CD (*p* = 0.249).

For echocardiography parameters, the change in SBP and DBP from baseline to peak were greater in the NCA-CD patients than in the CAD-CD patients (*p* = 0.011 and *p* = 0.001, respectively). Peak DBP and RPP were both significantly greater in NCA-CD patients (*p* = 0.044 and *p* = 0.007, respectively).

The overall frequency of symptoms reported during the test was not significantly different (*p* = 0.893). Limiting dyspnoea was experienced in 35.7% of patients with NCA-CD, compared to 6.7% of patients with CAD-CD (*p* = 0.080). There was no significant difference in the reporting of chest pain between the two groups (*p* = 0.089). After adjusting for severity, severe chest pain was reported more frequently in patients with CAD compared to NCA patients (53.3% vs. 14.3%, *p* = 0.050).

#### Hypertensive response to stress

Patients with NCA-CD but a hypertensive response to exercise (*n* = 12) were compared with patients who also elicited a hypertensive response but had a normal LV response to exercise (*n* = 37), as shown in Table 4.

**Table 4:** Patient characteristics and SBSE parameters in patients with a hypertensive response to exercise, comparing patients with nonobstructed coronary arteries and cavity dilatation (NCA-CD) to those with a normal left ventricular response to exercise (normal SBSE)

Variable	NCA-CD & hypertensive response (*n* = 12)	Normal SBSE & hypertensive response (*n* = 37)	*p* value
**Patient characteristics**			
Age (years)	60.5 ± 8	60.8 ± 10.8	*p* = 0.919
Females, % (n)	66.7 (8)	43.2 (16)	*p* = 0.196
BMI, kg/m^2^	27.8 ± 4.8	30.2 ± 5.1	*p* = 0.154
Antihypertensive medication, % (n)	58.3 (7)	62.2 (23)	*p* = 1.000
Type 2 diabetes, % (n)	16.7 (2)	16.2 (6)	*p* = 1.000
Dyslipidaemia, % (n)	33.3 (4)	37.8 (14)	*p* = 1.000
Smoker or ex-smoker, % (n)	58.3 (7)	40.5 (15)	*p* = 0.331
FH of CAD, % (n)	58.3 (7)	54.1 (20)	*p* = 1.000
PMH of anxiety or depression, % (n)	50 (6)	24.3 (9)	*p* = 0.148
**Echocardiography parameters**			
Resting HR	76 ± 14	82 ± 15	*p* = 0.220
Peak HR	137 ± 15	135 ± 13	*p* = 0.684
Change in HR	61 ± 14	53 ± 14	*p* = 0.102
Resting SBP	152 ± 32	152 ± 20	*p* = 1.000
Peak SBP	213 ± 26	219 ± 20	*p* = 0.475
Change in SBP	61 ± 34	67 ± 28	*p* = 0.588
Resting DBP	79 ± 15	85 ± 13	*p* = 0.231
Peak DBP	112 ± 15	110 ± 19	*p* = 0.711
Change in DBP	33 ± 20	25 ± 20	*p* = 0.244
Resting RPP	11,569 ± 3066	12,694 ± 3186	*p* = 0.287
Peak RPP	29,125 ± 4466	28,581 ± 5175	*p* = 0.728
Exercise time (mins: secs)	08:04 ± 03:02	07:43 ± 03:14	*p* = 0.736
Symptoms during test, % (n)	66.7 (8)	43.2 (16)	*p* = 0.196
Chest pain during test, % (n)	25 (3)	13.5 (5)	*p* = 0.386
Dyspnoea during test, % (n)	41.7 (5)	27 (10)	*p* = 0.473

Data are expressed as the mean ± standard deviation or as a proportion of the population (%), with n = the number of patients. SBSE: supine bicycle exercise stress echocardiography

## Discussion

This study is the first to assess the prevalence of LV cavity dilatation during SBSE in a large, unselected cohort and to explore the potential underlying mechanisms involved. Cavity dilatation was observed in 5.1% of patients who underwent SBSE. In approximately 50% of these cases, the dilatation was attributable to underlying obstructive CAD, and this subgroup showed a higher observed frequency of severe chest pain during exercise (*p* = 0.050). However, there were no consistent clinical or stress echocardiography predictors for distinguishing between normal and abnormal LV responses to exercise in the context of elevated BP at peak stress. Cavity dilatation during stress echocardiography has previously been found to be prognostically important, with associations to all-cause mortality in patients with CAD [18] and those with NCA [19].

The prevalence of LV cavity dilatation observed during SBSE is comparable to that reported in a previous study utilising treadmill stress echocardiography, where 2.2% of those presenting for stress echocardiography exhibited cavity dilatation in the absence of obstructive CAD [20], suggesting that the mode of exercise stress does not significantly influence the frequency of an abnormal LV response.

### CAD-associated cavity dilatation (CAD-CD) vs. non-CAD cavity dilatation (NCA-CD)

#### Patient characteristics

Although limited by small subgroup sizes, patients with NCA-CD tended to be female, have a greater change in SBP and DBP during exercise, and experienced dyspnoea during the test. Conversely, those with CAD-CD had a lower peak RPP and were more likely to experience severe chest pain during the test.

While the presence of CAD in this population is clinically important, and downstream anatomical testing is warranted for potential revascularisation or medical therapy, CAD does not appear to strongly influence long-term outcomes. In a large cohort study of patients presenting with abnormal stress echocardiography, long-term outcomes were similar in those with nonobstructive CAD compared to patients with obstructive CAD, with an adjusted likelihood of subsequent death over 2.4 years of follow-up of 1.04 for patients with stenoses < 50% versus those with > 50% stenoses [10]. A more recent study that found that patients with a reduction in LVEF at peak exercise had a 10-year incidence of heart failure hospitalisation of 17.6%, despite a negative coronary angiogram [19]. These findings highlight that maladaptive LV response in the absence of CAD is not a benign entity, suggesting that adverse outcomes are driven by alternative pathophysiological mechanisms that are not yet fully understood.

The preponderance of LV cavity dilatation in females in the absence of obstructive CAD has been documented in prior studies [11, 19, 21]. One proposed mechanism is coronary microvascular dysfunction (CMD), which occurs more frequently among peri- and postmenopausal women and may be linked to reduced circulating oestrogen levels [22, 23]. Supporting this hypothesis, hormone replacement therapy appears to protect against the development of CMD and associated chest pain, further implicating oestrogen deficiency as a plausible pathophysiological mechanism in this setting [24]. CMD may reflect several underlying abnormalities, including impaired vasodilation of arterioles [25], and can be estimated non-invasively using myocardial perfusion reserve (MPR). In one study of patients with symptoms suggestive of ischaemia but no obstructive CAD on invasive angiography, stress cardiac magnetic resonance imaging was used to calculate MPR [26]. This study revealed that 24% had definite CMD (MPR < 2), and an additional 35% had borderline CMD (MPR 2-2.4) [26]. These findings indicate CMD is likely highly prevalent amongst the NCA-CD population and may serve as a potential mechanism underlying LV cavity dilatation in this subgroup.

This phenomenon has been previously described in females with a low pre-test probability of CAD and has been characterised as a false positive stress echocardiography response, with a hypertensive response to exercise identified as the principal contributing factor in approximately one-third of cases [27]. In this setting, transient LV functional abnormalities have been attributed to excessive increases in RPP and afterload, resulting in a mismatch between myocardial oxygen supply and demand rather than flow-limiting epicardial coronary disease. Such haemodynamic effects may lead to reversible LV cavity enlargement and reductions in LVEF during stress, particularly in females with exaggerated BP responses. In our cohort, 49 patients (15%) without obstructive CAD had a hypertensive response to exercise; however only 12 of these patients (4%) exhibited associated LV cavity dilatation. Given that the majority of hypertensive responders demonstrate a normal LV response to exercise, the presence of cavity dilatation despite non-obstructive CAD may identify a distinct subgroup rather than representing a simple false positive finding and may reflect a higher-risk phenotype with the adverse outcomes reported in patients with NCA-CD [10].

Anxiety and depression may also contribute, as they have been associated with elevated plasma catecholamine levels [28–30] which could promote a maladaptive LV response to stress by promoting coronary vasospasm [31–33]. A higher prevalence of anxiety and depression in the NCA-CD cohort may therefore represent an important contributing factor. However no significant difference between groups was observed in this study, which may reflect the limited sample size.

#### Stress echocardiography

##### Blood pressure response

Although the peak SBP was similar between the two groups, patients with NCA-CD demonstrated a greater rise in SBP during exercise than patients with CAD-CD (*p* = 0.011). An exaggerated blood pressure response to exercise has been previously associated with reduced coronary flow velocity and may predict CMD [34]. Schumann et al. also found that prevalence of hypertension was significantly higher in patients with reduced MPR, consistent with definite CMD, in compared to those with a normal or borderline MPR [26]. However, it remains unclear why only a subset of patients with a hypertensive response in this study exhibited cavity dilatation, whereas others with a similar blood pressure profile did not. Our analysis did not identify any distinguishing clinical characteristics between these two subgroups.

Peak DBP was significantly higher in patients with NCA-CD than in those with CAD-CD. Patients with NCA-CD also had a significantly greater increase in DBP from resting to peak exercise. Usually, exercise induces a decrease in DBP due to systemic vasodilation [35]. An increase in DBP during exercise may suggest impaired ventricular relaxation. Previous studies have proposed that a rise in DBP of $\:\ge\:$15 mmHg from baseline to peak exercise represents an abnormal response and may be indicative of underlying CAD [36, 37]. Our study revealed the opposite - patients without obstructive CAD and cavity dilatation presented a greater increase in DBP. These discrepancies may reflect differences in study populations, as earlier studies were conducted over 40 years ago and involved predominantly male, high-risk cohorts, limiting their generalisability.

##### Symptoms during exercise

While chest pain incidence did not differ significantly between groups, patients with CAD-CD showed a higher observed frequency of severe chest pain during SBSE compared with NCA-CD patients. Conversely, there was a trend toward more frequent dyspnoea in the NCA-CD group. The mechanisms underlying these findings are uncertain but may be related to undiagnosed heart failure with preserved ejection fraction (HFpEF) in this subgroup. Diastolic dysfunction – particularly in individuals with a maladaptive LV response to exercise-induced hypertension – could contribute to these symptoms [38]. Prior studies in HFpEF patients reveal a disproportionate rise in pulmonary capillary wedge pressure relative to cardiac output in response to exercise, attributable to factors such as abnormal ventricular-arterial coupling and impaired stroke volume augmentation [38]. Further research, including invasive or advanced non-invasive evaluation of left atrial pressure during exercise in this cohort, would help clarify the pathophysiological basis of exertional dyspnoea in this population.

### Study limitations

While this study provides valuable insights, several limitations must be considered when interpreting the findings. Given that a maladaptive response to exercise occurs infrequently, the sample sizes were relatively small and therefore susceptible to random variability and bias, potentially making them less representative of the larger population. These small numbers are in keeping with other published studies in this area [38]. The retrospective nature of the study limits the ability to infer causality and although minimal, there is potential that missing data could lead to potential bias. It is also important to note that LV cavity dilatation and reduction in LVEF were assessed visually at peak exercise by an experienced echocardiographer rather than by quantitative volumetric measures, reflecting real-world clinical practice but potentially limiting reproducibility. Although volumetric methods would have allowed quantitative data analysis, it is highly time-consuming and subject to operator-dependent variability, limiting its feasibility and reproducibility in routine high workload exercise imaging.

During SBSE, symptom assessment was not standardised, with operator-dependant reporting of chest pain symptoms which limits the reproducibility of symptom-related findings. Finally, the study lacks longitudinal outcome data, precluding assessment of the prognostic significance of exercise-induced LV cavity dilatation; further studies evaluating clinical implications are warranted in this setting and should be a focus of further research.

## Conclusion

LV cavity dilatation during SBSE occurs in approximately 5% of patients and is attributable to obstructive CAD in about half of these cases, who showed a tendency toward more severe exertional chest pain. The other half of patients have either normal coronary arteries or non-obstructive CAD, commonly exhibiting a hypertensive blood pressure response to exercise and are predominantly female. This sub-group remains poorly understood, underscoring the need for further research into its underlying mechanisms and potential therapeutic strategies.

## Data Availability

All the data generated or analysed during this study are included in this published article.
